# Management of primary anterior shoulder dislocations: a narrative review

**DOI:** 10.1186/s40798-019-0203-2

**Published:** 2019-07-11

**Authors:** Andrew W. Hasebroock, Joseph Brinkman, Lukas Foster, Joseph P. Bowens

**Affiliations:** Creighton University School of Medicine, 2500 California Plaza, Omaha, NE 68105 USA

**Keywords:** Anterior shoulder dislocation, Anterior shoulder dislocation management, Anterior shoulder dislocation treatment, Anterior shoulder dislocation recurrence

## Abstract

**Abstract:**

The recurrence rate following acute anterior shoulder dislocations is high, particularly in young, active individuals. The purpose of this paper is to provide a narrative overview of the best available evidence and results with regards to diagnostic considerations, comorbidities, position of immobilization, surgical versus conservative management, and time to return to play for the management of primary anterior shoulder dislocations. Three independent reviewers performed literature searches using PubMed, MEDLINE, EMBASE, and Cochrane Central Register of Controlled Trials. Randomized controlled trials and systematic reviews meeting inclusion criteria from 1930 to April 2019 were appraised and discussed with the intent to consolidate the best available evidence with regards to lowering recurrence rates. A majority of studies support early surgical intervention for individuals between 21 and 30 years of age following primary shoulder dislocations, as this group is particularly susceptible to recurrence. Conservative treatment plans favor 1–3 weeks of immobilization in internal rotation, followed by rehabilitation. Surgical methods are associated with longer time to return to play, but lower recurrence rates. Return to play time is best determined on an individualized basis, when subjective and objective function of both shoulders is determined to be symmetric. This paper broadly summarizes the best available evidence for the management of primary anterior shoulder dislocations. There remains a need for randomized studies to determine ideal long-term treatment following conservative or surgical management, as general timelines for returning to play following injury remain vague.

**Level of evidence:**

IV, Narrative Review

## Key points

- If a patient is young and active, particularly under the age of 30, they are far more likely to dislocate the shoulder and, if not a young adolescent, will have superior long-term results with surgical management.
- Following surgical stabilization, or simple reduction, internal immobilization for 1–3 weeks is recommended.
- Specially designed rehab protocols have been shown to accelerate time to return to sport, but the decision to return to full activity should be based on symmetric range of motion and strength in comparison to the healthy shoulder.
- Optimal management involves approaching each case on an individual basis and relying on functional comparisons of strength, range of motion, and sport-specific requirements at scheduled check-ups to make important decisions concerning management and return to play.

## Background

The geometry of glenohumeral articulation permits great flexibility at the expense of intrinsic stability. This inherent instability makes the shoulder the most commonly dislocated joint in the body, which can lead to recurrent dislocations or subluxations [1]. In particular, young, active males under the age of 30 have an increased risk of recurrent instability [2, 3]. Nearly half (48.6%) of all shoulder dislocations occur in patients 15 to 29 years old, with the highest rate of recurrent dislocations (64%) found in those under age 30 and a male-to-female incidence rate ratio of 2.64 [3]. With evolving knowledge of this common injury, optimal management of primary anterior shoulder dislocations remains controversial.

The first question physicians will usually face following the onset of injury to an athlete is when the affected individual will return to competition. Traditionally, athletes with shoulder injuries are permitted to return to play when range of motion and strength of the affected side are comparable to the unaffected side. However, the extensive variability of injuries and specific individual differences, such as hand dominance, chronicity of injury, and age, make it difficult to estimate the exact timeline it will take a patient to reach this point. Compounding this issue is a lack of literature providing systematic guidelines for common traumatic shoulder injuries [4]. As a result, there may be large discrepancies among physicians on the optimal management and timeline to return to play following an anterior shoulder dislocation in an athlete.

Recurrent shoulder instability following a traumatic dislocation usually develops within the first 2 years of primary dislocation [5, 6]. Because the first 2 years following a primary anterior shoulder dislocation are crucial in long-term outcomes, understanding the optimal management following common anterior shoulder dislocations will assist both physicians and patients in deciding between courses of treatment [5, 7]. The purpose of this study, then, is to comprehensively consolidate existing literature and provide a guide for optimal evidence-based management of traumatic anterior shoulder dislocations. The lack of consensus in this area of study warrants the need for this review.

## Main text

### Methods

#### Literature search

Articles reviewed in this narrative overview were obtained from PubMed, MEDLINE, EMBASE, and Cochrane Central Register of Controlled Trials database searches dating from 1930 to April 2019. Our overall goal was to identify influencing variables and the magnitude of their effects on primary anterior shoulder dislocation recurrence rates. With that goal in mind, search terms “primary traumatic anterior shoulder dislocation” and “anterior shoulder dislocation management” were combined with the following injury and surgical specific search terms: primary, acute, first time, Bankart lesion, Hill-Sachs lesion, arthroscopic Bankart repair, conservative, immobilization, return to play, or return to sport.

#### Types of studies

Two types of studies were primarily included in the formation of this narrative overview: systematic reviews of anterior shoulder dislocations, and randomized controlled trials (RCTs) or quasi-randomized controlled trials of levels I, II, III, or IV evidence that have evaluated operative or non-operative treatment of primary anterior traumatic shoulder dislocations. This included studies investigating the outcomes of duration of immobilization, position of immobilization, or operational repairs. Anterior dislocations account for over 95% of shoulder dislocations [8], so we chose to study these in order to focus on a more common pathology. Additionally, Bankart lesions are present in 73–85% of anterior dislocation cases. [9] To address this, we included the most common surgery to repair these: arthroscopic Bankart repair (ABR). We also investigated the Latarjet procedure and open surgical repair of primary anterior shoulder dislocations. Finally, literature focused on specific rehabilitation protocols was determined to be out of the scope of this paper and better suited for a separate review. All studies considered followed ethical guidelines for human and animal rights.

#### Inclusion and exclusion criteria

Our overall inclusion criteria were the following:

1. Levels of evidence I, II, III, or IV
2. Randomized controlled trials or quasi-randomized studies, or systematic reviews
3. Primary anterior shoulder dislocation studies
4. Non-operative management: duration or position of immobilization studied
5. Operative management: arthroscopic or open
6. Recurrences of instability (or recurrence rate) recorded

Exclusion criteria included the following:

1. Specialized or aggressive rehabilitation protocol
2. Posterior shoulder dislocation studies

### Results

#### Diagnosis

Anterior shoulder dislocations are often clinically diagnosed given their classic appearance. Patients normally present with their arm adducted and internally rotated, showing a loss of normal deltoid contour [8]. A posterior sulcus or glenohumeral void may be visible, and the humeral head may be palpable anteriorly. Radiographs can be used to confirm the diagnosis, as well as to visualize concurrent damage to bone. In general, radiographs before reducing the shoulder are not necessary unless one of the following three conditions is met: age over 40, first-time dislocation, traumatic mechanism of injury. If these three factors are negative, there is a negative predictive value of 96.6% for associated fracture [10]. Standard radiographs to assist with initial diagnoses, or in post-reduction assessment, are the following: anteroposterior views in neutral, external, and internal rotation, a lateral, or “Y,” view in the scapular plane, and an axillary view [11]. Computerized tomography (CT) scans are not routinely used initially, except to better assess for bone loss in first-time dislocators if surgery is required or a CT angiogram for possible vascular injury. MRI is best for soft tissue pathology, such as damage to the labrum, axillary nerve, or shoulder capsule, but is also infrequently used as history and physical exam provide most of the essential information [11].

#### Associated complications

##### Bone involvement

Evaluating bone injury before reduction is necessary as humeral fractures can cause complications and further damage. A relatively common injury associated with anterior shoulder dislocations involves the posterolateral head of the humerus impacting the anteroinferior glenoid, causing a cortical depression in the posterolateral head of the humerus, known as a Hill-Sachs lesion [12]. The prevalence of Hill-Sachs lesions was found to be 54% in a large-scale study of anterior dislocations [13]. In similar fashion, a bony Bankart lesion occurs when the anterior rim of the inferior glenoid is damaged by the shifting head of the humerus. The prevalence of Bankart lesions in anterior shoulder dislocations has been reported as approximately 73% in two studies [14, 15]. A defect of over 20% of the area is considered “critical,” and to be managed surgically. Detecting these lesions is of value given their association with an increased risk of recurrent dislocation. However, it has been demonstrated that instability severity index scores below 7 are not useful in predicting increased recurrence risk [16]. In terms of vascular injuries, shoulder dislocations with concomitant fractures are highly associated as only 1% of patients with vascular pathology show no bone injury [17]. Considering these associations, evaluating bone damage in shoulder dislocations is important in determining appropriate management strategies.

##### Nerve involvement

Nerve injuries also occur in association with shoulder dislocations. Axonal loss following anterior dislocation has been found to be 45–48% with notable risk factors of age, bruising, and fractures [18, 19]. The axillary nerve is most commonly affected and detected clinically with functional loss of deltoid movement and sensation over lateral shoulder. Less frequently, the brachial plexus can be injured as a result of lateral traction produced by the dislocation. Patterns of damage depend on humeral position at the time of dislocation. Abduction and internal rotation can cause damage to all cords while extension of the elbow and wrist is associated with medial cord injury. The posterior and medial cords can both be impacted by dislocation with elbow flexion [20].

##### Vascular involvement

Vascular injuries are infrequent but emergent complications of shoulder dislocations. Axillary artery transection, a rare but major complication of shoulder dislocation, portends high morbidity if not properly recognized [21]. The incidence of arterial injuries with dislocations has been reported as 2% [22]. Although vascular issues are more common in dislocations of older patients or those with atherosclerosis, they can occur in individuals of any age [18]. In a review of 90 such cases, the morbidity was found to be 50% when reduction was performed weeks after dislocation [23]. Detecting vascular injury commonly involves diminished pulses and protruding axillary hematoma, although collateral circulation may provide capillary filling despite present arterial injury [24]. In cases with either diminished pulses or decreased skin temperature, an angiogram is recommended to properly evaluate vascular state.

##### Rotator cuff tears

Soft tissue abnormalities of the labrum, glenoid, or tendons are commonly associated with anterior shoulder dislocations. During dislocation, the humeral head may avulse and form a pocket within the anterior glenoid and labrum, known as a Bankart lesion [25]. The frequency of anterior shoulder dislocations with concomitant Bankart lesions has been shown to be as high as 83%, with a rate of 78.2% in acute dislocations, and Bankart and/or anterior labroligamentous periosteal sleeve avulsion (ALPSA) lesions in up to 97.11% of patients with chronic instability [26]. Rotator cuff tears also occur alongside dislocations at a frequency ranging from 7 to 32%, with older individuals more commonly affected [27–29]. These tears, due to prognostication of continued shoulder instability, require prompt attention, and can be confirmed with MRI if suspected on physical exam.

#### Management

##### Reduction

Timely management of anterior shoulder dislocations is absolutely essential for optimal patient outcomes, as there is elevated risk of unstable reduction if the shoulder is left untreated for over 24 h from initial injury [13]. Early reduction also leads to lower risk of muscle spasm and damaging manipulation of neurovascular structures within the shoulder [30]. While there is clear consensus regarding the timeline of anterior dislocated shoulder reduction, the optimal method of reduction is less obvious. There are over 20 different methods of shoulder reduction with variations in traction, leverage, and scapular manipulation; however, no optimal reduction method has been established [31, 32]. A clinician’s choice of reduction method is primarily based on personal preference and the ability of the patient to maintain his or her shoulder in the appropriate position [32–34]. In view of procedural sedation for dislocation reduction, a combination of a narcotic and benzodiazepine with or without the addition of propofol is most commonly used [35]. Remifentanil was recently shown to have equal analgesic efficacy and shorter onset compared to the combination of propofol and fentanyl but also exhibited significantly higher rates of apnea [36]. Inhaled methoxyflurane, when used in the emergency department for shoulder dislocations, was recently found to have a shorter recovery time and be of equal efficacy to propofol [37].

##### Immobilization

Several studies have investigated optimal immobilization techniques for the management of anterior shoulder dislocations. One such study demonstrated the risk of dislocation recurrence was not influenced by the chosen form of immobilization by finding that patients who wore a standard sling until they felt comfortable without it showed similarly proficient long-term outcomes compared to patients with formal forms of immobilization [13]. A meta-analysis found immobilization for longer than 1 week following anterior shoulder dislocation does not improve the risk of recurrence. The same study also demonstrates slightly decreased rates of dislocation recurrence with external rotation immobilization over internal rotation [38]. A 2018 study of 50 patients found that external rotation showed a significant improvement over internal rotation for recurrent dislocations in the 20–40 age subgroup [39]. This may be due to less separation of the torn labrum and increased labrum-glenoid contact force in Bankart lesions when the affected shoulder is externally fixated [40, 41]. While these results are promising, external rotation immobilization can be a more awkward position for patients, posing temporary difficulties with daily activities. This should be taken into consideration when choosing the best form of immobilization for a patient.

Reports of external rotation are not unanimously favorable. A recent literature review of the position of immobilization after first-time traumatic anterior shoulder dislocations found that external rotation posed no superiority to internal rotation [42]. Although external rotation did result in better coaptation of the labrum and glenoid fossa, there were no significant differences in dislocation recurrence rates or patient quality of life. It is speculated that external rotation immobilization may be the method of choice for patients with a specific labroligamentous injury with anterior shoulder dislocation, but research-supported conclusions are yet to be determined [42]. It has been suggested external rotation immobilization should only be considered in highly motivated, compliant patients who are informed of the discomfort and possible difficulties with everyday tasks while the brace is being used. Finally, high-demand patients, including professional athletes, should receive MRI evaluation before immobilization for any potential soft tissue injuries, such as a subscapularis muscle tear, which would eliminate the possibility for external rotation immobilization [42].

##### Surgical treatment

Management strategies for dislocations often include surgical options. For patients under 30, non-surgical treatment has been associated with significantly higher rates of recurrent dislocation outside of young adolescents [9, 43]. One study, over 10 years, determined that conservatively managed patients exhibited a 62% recurrence rate compared with 9% in surgically repaired patients [9]. Furthermore, arthroscopic surgical stabilization offers better shoulder mobility, satisfaction, and quicker return to activity time [44]. Surgical repair is an appealing option for high-risk patients who have experienced traumatic anterior shoulder dislocation, are between the ages of 21–30 years, and who participate in high-risk sports [45].

Much has been published about the surgical treatment options for shoulder dislocation. A review of 655 articles on surgery for shoulder instability found that 10 out of 31 procedures were given grade A or B recommendations [46]. Those given grade A in favor of recommendation included open Bankart, arthroscopic Bankart, and the Latarjet procedures. For Bankart lesions, surgical repair has shown high success in preventing recurrent dislocations with low surgical morbidity and is suggested to be superior to conservative immobilization [47, 48]. Recurrent instability after arthroscopic Bankart repair has been reported to be as low as 8.1% and associated with younger patients, bilateral involvement, and closed reduction prior to repair [43]. Overall failure rates of Bankart repair, pooled from a meta-analysis of 12 studies, was found to be 13.7% [49]. A study analyzing the success of this procedure found failures commonly arise due to technical errors or improper patient selection. Taking this into consideration, some clinicians suggest surgical Bankart repair should be reserved for patients with unidirectional, post-traumatic, anterior instability, and well-developed ligamentous tissue due to failures [50]. The Latarjet procedure involves transplant of the coracoid process to the scapular neck to treat recurrent dislocation and has demonstrated excellent long-term clinical outcomes and return to sport rate [51, 52]. Recurrent instability is reported to be as low as 0–5.4% [53, 54]. Most complications of the procedure can be avoided with proper surgical technique but further research is underway in areas related to adverse outcomes including graft position and osteolysis [55].

In terms of surgical strategy, there is an ongoing discussion as to whether open or arthroscopic repair is superior. Long-term investigations have found a higher recurrence of instability in arthroscopic repair compared to open techniques [56, 57]. However, this is contended by randomized clinical trials, investigations with adolescents, and long-term follow-up studies that found no significant difference in clinical outcomes between the two surgical approaches [58–60]. In regard to complications, a review of 56 studies considering 4362 procedures noted complication rates of 1.6% and 6.2% for arthroscopic and open techniques, respectively [61]. Most common complications included unspecified hardware problems, postoperative stiffness, nerve injury, nonunion, and infection. Subsequent evaluation is warranted before either open or arthroscopic approach can be definitively deemed more advantageous [8].

#### Prognosis

##### Re-dislocation

Re-dislocation of the glenohumeral joint is very common even after appropriate treatment, with some studies reporting recurrence rates exceeding 70% [62–64]. However, rates of recurrence vary greatly, and patient age seems to play a significant role in the determination of that rate. One study of 15,246 anterior shoulder dislocations found an overall recurrence of 28.7%. Interestingly, the dislocation recurrence rate was higher in soldiers under the age of 40 [65]. Another study of 154 anterior shoulder dislocations found a similar trend, with a 68% recurrence rate in patients under 20 years old, 54% in patients under 30, and only 12% in patients over 30 [66]. A more recent study focused only on those who re-dislocated within 1 year of the initial event and found a recurrence rate of 46% in 128 participants [67].

The commonality of recurrent anterior shoulder dislocations can be attributed to the shoulder anatomy deformities present following initial dislocation. Such injury-caused deformities include abnormal laxity of the joint capsule and surrounding muscles, deformities of the head of the humerus, and contracture of the muscles surrounding the glenohumeral joint [13, 67, 68]. Of note, greater tuberosity fractures have been shown to decreased the risk of recurrent instability in patients who obtained the injury in first-time traumatic anterior shoulder dislocations [68]. Subsequent anterior shoulder dislocations increase the risk of glenoid bone loss, exacerbating the already existent shoulder deformities present after initial injury [69]. An evaluation of 714 athletes found a glenoid bone loss of 6.8% after a first-time anterior shoulder instability event and a total calculated glenoid bone loss of 22.8% in the setting of recurrent instability [70]. A primary study evaluating recurrent dislocations in cases of glenoid bone loss found recurrence rates similar to the rest of the literature, with a 27% rate in patients over 30 and a 72% rate in patients under 23 years old [13]. In addition, more recent studies, including a systematic review and meta-analysis, found that younger patient age, male sex, glenohumeral joint hyperlaxity, higher activity levels, increased pain, and higher levels of reinjury fear also increase the risk of dislocation recurrence [13, 67]. A separate investigation observed recurrent dislocations in view of anatomical factors and found that increases in the humeral containing angle and glenoid height-to-width ratio were significant risk factors [71].

##### Return to sport following conservative management

The timeline from dislocation to returning to activity is a key concern for patients and will commonly be the first question encountered by the diagnosing physician. Interestingly, most clinical recommendations in this regard are based on individual anecdotal experience instead of clear guidelines [8]. Most treatment regimens aimed at an efficient return recommend an initial short period of immobilization in a simple sling between 1 and 3 weeks [72]. For returning to activity, some clinicians advise the patient that return is permissible when range of motion and strength are near normal [32]. A study by Watson et al. agrees with this, noting the general consensus that the patient should be pain free with symmetric scapular strength before returning, generally occuring within 2–3 weeks [8]. However, this notion has been challenged by a study that showed patients who returned before 6 weeks had significantly poorer outcomes than patients who waited over 6 weeks to return [73]. While there are no current evidence-based parameters on goal rotator cuff strengths before return to sport, one recent study found weakness in internal and external rotator strength was associated with recurrent anterior shoulder instability [74]. This suggests symmetric rotator cuff strength between shoulders may be a sensible recommendation before allowing full return to sport following conservative management.

##### Return to sport following surgery

Patients are typically able to return to sport anywhere from 4 to 6 months following surgical correction of an anterior shoulder dislocation and most are able to achieve pre-injury activity level [75]. An investigation of 58 football players with surgical intervention of shoulder dislocations found that 98.7% were able to return to play for at least 1 year without subsequent injury [76]. Another study of 57 athletes in various sports found that all study participants were able to return to play and 66% of them stated surgical repair improved their shoulder functionality in their respective sport compared to preoperative condition [77]. Of 51 baseball players who underwent arthroscopic Bankart repair, the average return to play was 8.4 months with those in non-throwing positions demonstrating the best results [78]. This study also found 90% of athletes were able to participate in at least one game with follow-up duration set at 24 months.

Appropriate time for return to play is determined by symmetrical abduction and external rotation of the glenohumeral joint [6, 64, 76]. Timeline for post-op therapy varies depending on surgeon preference, but the following multiphase approach is most commonly seen. Phase 1 includes sling immobilization for 4 weeks with isometric contractions. Phase 2 includes limiting active range of motion of the glenohumeral joint to 45° of external rotation for 4 weeks. Phase 3 includes allowing full active range of motion while using resistance and plyometric training to strengthen the joint, restore full range of motion, and improve proprioceptive feedback before finally allowing the patient to return to full activity. Overall, the currently used postoperative anterior shoulder dislocation rehabilitation timelines seem to work very well [76–78].

## Conclusions

Achieving the best long-term results when managing primary anterior shoulder dislocations requires a systematic series of decisions. First, the proper diagnosis must be made, as well as detection of any comorbidities. X-ray is the best initial imaging when reduction of the joint is necessary. Additional studies should be added if comorbidities such as vascular, nerve, or soft tissue damage are suspected. Reduction using the clinician’s preferred method and appropriate pain control is the next best step, followed by post-reduction films to ensure proper positioning. The next vital information is risk stratification, with two of the most important predictors of recurrence being age and activity level.

If a patient is young and active, particularly under the age of 30, they are far more likely to redislocate the shoulder. For this population, we suggest discussing the risks and benefits of conservative versus surgical approaches with patients, as surgical options have been shown to have superior long-term results in this patient population outside of young adolescents. Additionally, surgical approaches have a more predictable course of recovery, but carry larger risks. Decisions on surgical approach should focus on comorbidities present, as well. Conservative management appears to be the best initial management for elderly patients or young patients not involved in demanding overhead activities.

Following surgical stabilization, or simple reduction, internal immobilization for 1–3 weeks is recommended. Evidence supporting external immobilization has not been reproduced and is oftentimes uncomfortable for patients. Physical therapy and a series of exercises should always be included in long-term management, with 3 months as the preferred time course. Specially designed rehab protocols have been shown to accelerate time to return to sport, but the decision to return to full activity should be based on symmetric range of motion and strength in comparison to the healthy shoulder. In the case of a competitive athlete, they are able to return to play within 2 to 3 weeks, but there is a high risk of recurrent instability. Therefore, this is not recommended if the goal is a lower recurrence rate. Additional randomized controlled trials are necessary to further explore optimal long-term management. Currently, recommendations for unrestricted return to play remain broad and generalized, as opposed to a specific timeline. At this time, then, it is best to approach each case on an individual basis and rely on functional comparisons of strength, range of motion, and sport-specific requirements at scheduled check-ups to make these important decisions.

## Limitations

An important limitation of a narrative overview of this kind is generalizability of the groups of patients upon which each study focused. In almost every study reviewed, the percentage of male patients exceeds 50%, so there is not an even distribution of male to female patients, and males have been associated with a higher rate of recurrence [3]. Additionally, most studies reviewed in the operative repair section had a mean age below age 30, and younger age is the largest predictor of recurrent dislocations [2, 3, 40, 45]. Heterogeneity is a great issue for generating conclusions based on available literature because primary outcomes between the studies varied. We must acknowledge, then, that although specialized rehabilitation programs were excluded, those following similar, standard shoulder strengthening rehabilitation were included. It is impossible to know the compliance of each of these patients and their exact return as it was not always reported, nor do we know how strict the rehabilitation schedules were enforced. Other possible confounding factors for the studies summarized included surgeon skill level, presence of publication bias, small individual study sizes, severity of injuries, significant variability in methods of injury, and unreported variation in shoulder capsular damage.

## Data Availability

Data sharing not applicable to this article as no datasets were generated or analyzed during the current study.

## Abbreviations

ABR: Arthroscopic Bankart Repair
RCT: Randomized controlled trial

## References

[1] Kazár B, Relovszky E (1969). Prognosis of primary dislocation of the shoulder. Acta Orthop Scand..

[2] Heidari K, Asadollahi S, Vafaee R, Barfehei A, Kamalifar H, Chaboksavar ZA (2014). Immobilization in external rotation combined with abduction reduces the risk of recurrence after primary anterior shoulder dislocation. J Shoulder Elbow Surg..

[3] Zacchilli MA, Owens BD (2010). Epidemiology of shoulder dislocations presenting to emergency departments in the United States. J Bone Joint Surg Am..

[4] Marans HJ, Angel KR, Schemitsch EH, Wedge JH (1992). The fate of traumatic anterior dislocation of the shoulder in children. J Bone Joint Surg Am..

[5] Pavlik A, Csépai D, Hidas P, Bánóczy A (1996). Sports ability after Bankart procedure in professional athletes. Knee Surg Sports Traumatol Arthrosc..

[6] Owens BD, DeBerardino TM, Nelson BJ, Thurman J, Cameron KL, Taylor DC (2009). Long-term follow-up of acute arthroscopic Bankart repair for initial anterior shoulder dislocations in young athletes. Am J Sports Med..

[7] McGahan PJ, Fronek J, Hoenecke HR, Keefe D (2014). The use of an orthopaedic rating system in major league baseball. Sports Health..

[8] Watson S, Allen B, Grant JA (2016). A clinical review of return-to-play considerations after anterior shoulder dislocation. Sports Health..

[9] Jakobsen BW, Johannsen HV, Suder P, Søjbjerg JO (2007). Primary repair versus conservative treatment of first-time traumatic anterior dislocation of the shoulder: a randomized study with 10-year follow-up. Arthroscopy..

[10] Émond M, Le Sage N, Lavoie A, Rochette L (2004). Clinical factors predicting fractures associated with an anterior shoulder dislocation. Acad Emerg Med.

[11] Pollock RG, Bigliani LU (1993). Recurrent posterior shoulder instability: diagnosis and treatment. Clin Orthop Relat Res.

[12] Hill HA, Sachs MD (1940). The grooved defect of the humeral head: a frequently unrecognized complication of dislocations of the shoulder joint. Radiology..

[13] Hovelius L, Augustini BG, Fredin H, Johansson O, Norlin R, Thorling J (1996). Primary anterior dislocation of the shoulder in young patients. A ten-year prospective study. J Bone Joint Surg Am..

[14] Widjaja AB, Tran A, Bailey M, Proper S (2006). Correlation between Bankart and Hill-Sachs lesions in anterior shoulder dislocation. ANZ J Surg..

[15] Horst K, Von Harten R, Weber C, Andruszkow H, Pfeifer R, Dienstknecht T, et al. Assessment of coincidence and defect sizes in Bankart and Hill–Sachs lesions after anterior shoulder dislocation: a radiological study. Br J Radiol [Internet]. 2014 [cited 2019 Apr 24];87. Available from: https://www.ncbi.nlm.nih.gov/pmc/articles/PMC4064539/10.1259/bjr.20130673 PMC4064539 24452107

[16] Ruiz Ibán MA, Asenjo Gismero CV, Moros Marco S, Ruiz Díaz R, Del Olmo HT, Del Monte BG, et al. Instability severity index score values below 7 do not predict recurrence after arthroscopic Bankart repair. Knee Surg Sports Traumatol Arthrosc. 2019. Available from: https://link.springer.com/article/10.1007%2Fs00167-019-05471-w 10.1007/s00167-019-05471-w 30955072

[17] Sparks SR, DeLaRosa J, Bergan JJ, Hoyt DB, Owens EL (2000). Arterial injury in uncomplicated upper extremity dislocations. Ann Vasc Surg..

[18] Visser CP, Coene LN, Brand R, Tavy DL (1999). The incidence of nerve injury in anterior dislocation of the shoulder and its influence on functional recovery. A prospective clinical and EMG study. J Bone Joint Surg Br..

[19] de Laat EA, Visser CP, Coene LN, Pahlplatz PV, Tavy DL (1994). Nerve lesions in primary shoulder dislocations and humeral neck fractures. A prospective clinical and EMG study. J Bone Joint Surg Br..

[20] Cutts S, Prempeh M, Drew S (2009). Anterior shoulder dislocation. Ann R Coll Surg Engl..

[21] Magister S, Bridgforth A, Yarboro S (2018). Axillary artery injury following closed reduction of an age-indeterminate anterior glenohumeral dislocation. J Orthop Case Rep..

[22] Stayner LR, Cummings J, Andersen J, Jobe CM (2000). Shoulder dislocations in patients older than 40 years of age. Orthop Clin North Am..

[23] Calvet J, Leroy M, Lacroix L (1942). Luxations de l’epaule et lesions vasculaires. J Chir..

[24] Maweja S, Sakalihasan N, Van Damme H, Limet R (2002). Axillary artery injury secondary to anterior shoulder dislocation: report of two cases. Acta Chir Belg..

[25] Bankart ASB (1938). The pathology and treatment of recurrent dislocation of the shoulder-joint. Br J Surg.

[26] Yiannakopoulos CK, Mataragas E, Antonogiannakis E (2007). A comparison of the spectrum of intra-articular lesions in acute and chronic anterior shoulder instability. Arthroscopy..

[27] Imhoff AB, Ansah P, Tischer T, Reiter C, Bartl C, Hench M (2010). Arthroscopic repair of anterior-inferior glenohumeral instability using a portal at the 5:30-o’clock position: analysis of the effects of age, fixation method, and concomitant shoulder injury on surgical outcomes. Am J Sports Med..

[28] Simank H-G, Dauer G, Schneider S, Loew M (2006). Incidence of rotator cuff tears in shoulder dislocations and results of therapy in older patients. Arch Orthop Trauma Surg..

[29] Berbig R, Weishaupt D, Prim J, Shahin O (1999). Primary anterior shoulder dislocation and rotator cuff tears. J Shoulder Elbow Surg..

[30] Christofi T, Kallis A, Raptis DA, Rowland M, Ryan J (2007). Management of shoulder dislocations. Trauma..

[31] Alkaduhimi H, van der Linde JA, Flipsen M, van Deurzen DFP, van den Bekerom MPJ (2016). A systematic and technical guide on how to reduce a shoulder dislocation. Turk J Emerg Med..

[32] Kuhn JE (2006). Treating the initial anterior shoulder dislocation--an evidence-based medicine approach. Sports Med Arthrosc..

[33] Doshi D, Firke R (2014). A new patient-controlled technique for shoulder relocation in emergency departments. Am J Case Rep..

[34] Riebel GD, McCabe JB (1991). Anterior shoulder dislocation: a review of reduction techniques. Am J Emerg Med..

[35] Hatamabadi HR, Arhami Dolatabadi A, Derakhshanfar H, Younesian S, Ghaffari Shad E. Propofol versus midazolam for procedural sedation of anterior shoulder dislocation in emergency department: a randomized clinical trial. Trauma Mon [Internet]. 2015 [cited 2019 Apr 24];20. Available from: https://www.ncbi.nlm.nih.gov/pmc/articles/PMC4538724/10.5812/traumamon.13530 PMC4538724 26290851

[36] Monsef Kasmaee V, Zia Zibari SM, Aghajani NM (2019). Remifentanil versus propofol/fentanyl combination in procedural sedation for dislocated shoulder reduction; a clinical trial. Arch Acad Emerg Med..

[37] Umana E, Kelliher JH, Blom CJ, McNicholl B. Inhaled methoxyflurane for the reduction of acute anterior shoulder dislocation in the emergency department. CJEM. 2019:1–5. 10.1017/cem.2018.493 30739629

[38] Smith BI, Bliven KCH, Morway GR, Hurbanek JG (2015). Management of primary anterior shoulder dislocations using immobilization. J Athl Train..

[39] Murray J-C, Leclerc A, Balatri A, Pelet S. Immobilization in external rotation after primary shoulder dislocation reduces the risk of recurrence in young patients. A randomized controlled trial. Orthop Traumatol Surg Res. 2018.Available from: https://www.sciencedirect.com/science/article/pii/S1877056818303475?via%3Dihub 10.1016/j.otsr.2018.10.007 30502026

[40] Itoi E, Sashi R, Minagawa H, Shimizu T, Wakabayashi I, Sato K (2001). Position of immobilization after dislocation of the glenohumeral joint. A study with use of magnetic resonance imaging. J Bone Joint Surg Am..

[41] Miller BS, Sonnabend DH, Hatrick C, O’leary S, Goldberg J, Harper W (2004). Should acute anterior dislocations of the shoulder be immobilized in external rotation? A cadaveric study. J Shoulder Elbow Surg..

[42] Gutkowska O, Martynkiewicz J, Gosk J (2017). Position of immobilization after first-time traumatic anterior glenohumeral dislocation: a literature review. Med Sci Monit..

[43] Mahure SA, Mollon B, Capogna BM, Zuckerman JD, Kwon YW, Rokito AS (2018). Risk factors for recurrent instability or revision surgery following arthroscopic Bankart repair. Bone Joint J..

[44] Kirkley A, Werstine R, Ratjek A, Griffin S (2005). Prospective randomized clinical trial comparing the effectiveness of immediate arthroscopic stabilization versus immobilization and rehabilitation in first traumatic anterior dislocations of the shoulder: long-term evaluation. Arthroscopy..

[45] Kralinger FS, Golser K, Wischatta R, Wambacher M, Sperner G (2002). Predicting recurrence after primary anterior shoulder dislocation. Am J Sports Med..

[46] Glazebrook H, Miller B, Wong I. Anterior shoulder instability: a systematic review of the quality and quantity of the current literature for surgical treatment. Orthop J Sports Med [Internet]. 2018 [cited 2019 Apr 24];6. Available from: https://www.ncbi.nlm.nih.gov/pmc/articles/PMC6243418/10.1177/2325967118805983 PMC6243418 30480013

[47] Wen DY (1999). Current concepts in the treatment of anterior shoulder dislocations. Am J Emerg Med..

[48] Bock J, Buckup J, Reinig Y, Zimmermann E, Colcuc C, Hoffmann R (2018). The arthroscopic Bankart repair procedure enables complete quantitative labrum restoration in long-term assessments. Knee Surg Sports Traumatol Arthrosc..

[49] Adam M, Attia AK, Alhammoud A, Aldahamsheh O, Al Ateeq Al Dosari M, Ahmed G (2018). Arthroscopic Bankart repair for the acute anterior shoulder dislocation: systematic review and meta-analysis. Int Orthop.

[50] Warner JPJ, Miller MD, Marks P, Fu FH (1995). Arhtroscopic bankart repair with the suretac device. part I: clinical observations. Arthroscopy..

[51] Latarjet M (1954). Treatment of recurrent dislocation of the shoulder. Lyon Chir..

[52] Hurley ET, Jamal MS, Ali ZS, Montgomery C, Pauzenberger L, Mullett H (2019). Long-term outcomes of the Latarjet procedure for anterior shoulder instability: a systematic review of studies at 10-year follow-up. J Shoulder Elbow Surg..

[53] Carol EJ, Falke LM, Kortmann JH, Roeffen JF, van Acker PA (1985). Bristow-Latarjet repair for recurrent anterior shoulder instability; an eight-year study. Neth J Surg..

[54] Dossim A, Abalo A, Dosseh E, Songne B, Ayite A, Gnandi-Pio F (2008). Bristow-Latarjet repairs for anterior instability of the shoulder: clinical and radiographic results at mean 8.2 years follow-up. Chir Main..

[55] Gupta A, Delaney R, Petkin K, Lafosse L (2015). Complications of the Latarjet procedure. Curr Rev Musculoskelet Med..

[56] Freedman KB, Smith AP, Romeo AA, Cole BJ, Bach BR (2004). Open Bankart repair versus arthroscopic repair with transglenoid sutures or bioabsorbable tacks for Recurrent Anterior instability of the shoulder: a meta-analysis. Am J Sports Med..

[57] Neviaser AS, Benke MT, Neviaser RJ (2015). Open Bankart repair for revision of failed prior stabilization: outcome analysis at a mean of more than 10 years. J Shoulder Elbow Surg..

[58] Bottoni CR, Smith EL, Berkowitz MJ, Towle RB, Moore JH (2006). Arthroscopic versus open shoulder stabilization for recurrent anterior instability: a prospective randomized clinical trial. Am J Sports Med..

[59] Shymon SJ, Roocroft J, Edmonds EW (2015). Traumatic anterior instability of the pediatric shoulder: a comparison of arthroscopic and open bankart repairs. J Pediatr Orthop..

[60] Aboalata M, Plath JE, Seppel G, Juretzko J, Vogt S, Imhoff AB (2017). Results of arthroscopic Bankart repair for anterior-inferior shoulder instability at 13-year follow-up. Am J Sports Med..

[61] Williams HLM, Evans JP, Furness ND, Smith CD. It’s not all about redislocation: a systematic review of complications after anterior shoulder stabilization surgery. Am J Sports Med. 2018. 10.1177/0363546518810711 10.1177/0363546518810711 30525905

[62] Rowe CR (1956). Prognosis in dislocations of the shoulder. J Bone Joint Surg Am..

[63] Wheeler JH, Ryan JB, Arciero RA, Molinari RN (1989). Arthroscopic versus nonoperative treatment of acute shoulder dislocations in young athletes. Arthroscopy..

[64] Bottoni CR, Wilckens JH, DeBerardino TM, D’Alleyrand J-CG, Rooney RC, Harpstrite JK (2002). A prospective, randomized evaluation of arthroscopic stabilization versus nonoperative treatment in patients with acute, traumatic, first-time shoulder dislocations. Am J Sports Med..

[65] Kardouni JR, McKinnon CJ, Seitz AL (2016). Incidence of shoulder dislocations and the rate of recurrent instability in soldiers. Med Sci Sports Exerc..

[66] Vermeiren J, Handelberg F, Casteleyn PP, Opdecam P (1993). The rate of recurrence of traumatic anterior dislocation of the shoulder. A study of 154 cases and a review of the literature. Int Orthop..

[67] Olds MK, Ellis R, Parmar P, Kersten P (2019). Who will redislocate his/her shoulder? Predicting recurrent instability following a first traumatic anterior shoulder dislocation. BMJ Open Sport Exerc Med.

[68] Olds M, Ellis R, Donaldson K, Parmar P, Kersten P (2015). Risk factors which predispose first-time traumatic anterior shoulder dislocations to recurrent instability in adults: a systematic review and meta-analysis. Br J Sports Med..

[69] Milano G, Grasso A, Russo A, Magarelli N, Santagada DA, Deriu L (2011). Analysis of risk factors for glenoid bone defect in anterior shoulder instability. Am J Sports Med..

[70] Dickens JF, Slaven SE, Cameron KL, Pickett AM, Posner M, Campbell SE (2019). Prospective evaluation of glenoid bone loss after first-time and recurrent anterior glenohumeral instability events. Am J Sports Med..

[71] Hong J, Huang Y, Ma C, Qu G, Meng J, Wu H (2018). Risk factors for anterior shoulder instability: a matched case-control study. J Shoulder Elbow Surg..

[72] Owens BD, Dickens JF, Kilcoyne KG, Rue J-PH (2012). Management of mid-season traumatic anterior shoulder instability in athletes. J Am Acad Orthop Surg..

[73] Simonet WT, Cofield RH (1984). Prognosis in anterior shoulder dislocation. Am J Sports Med..

[74] Edouard P, Degache F, Beguin L, Samozino P, Gresta G, Fayolle-Minon I (2011). Rotator cuff strength in recurrent anterior shoulder instability. J Bone Joint Surg Am..

[75] Clesham K, Shannon FJ. Arthroscopic anterior shoulder stabilisation in overhead sport athletes: 5-year follow-up. Ir J Med Sci. 2019. 10.1007/s11845-019-01986-w 30771139

[76] Pagnani MJ, Dome DC (2002). Surgical treatment of traumatic anterior shoulder instability in american football players. J Bone Joint Surg Am..

[77] Plath JE, Feucht MJ, Saier T, Minzlaff P, Seppel G, Braun S (2015). Sporting activity after arthroscopic Bankart repair for chronic glenohumeral instability. Arthroscopy..

[78] Park J-Y, Lee J-H, Oh K-S, Chung SW, Lim J-J, Noh YM (2019). Return to play after arthroscopic treatment for shoulder instability in elite and professional baseball players. J Shoulder Elbow Surg..

